# Increased Risk of Cutaneous and Systemic Infections in Atopic Dermatitis—A Cohort Study

**DOI:** 10.1016/j.jid.2017.01.030

**Published:** 2017-06

**Authors:** Sinéad M. Langan, Katrina Abuabara, Sarah E. Henrickson, Ole Hoffstad, David J. Margolis

**Affiliations:** 1 Faculty of Epidemiology & Population Health, London School of Hygiene and Tropical Medicine, London, UK; 2 Program for Clinical Research, Department of Dermatology, University of California San Francisco (UCSF), San Francisco, California, USA; 3 The Children’s Hospital of Philadelphia, Division of Allergy Immunology and Department of Microbiology, University of Pennsylvania, Philadelphia, Pennsylvania, USA; 4 Department of Biostatistics and Epidemiology, University of Pennsylvania Perelman School of Medicine, Philadelphia, Pennsylvania, USA

**Keywords:** AD, atopic dermatitis

To the Editor

Atopic dermatitis (AD, also known as atopic eczema or eczema), is characterized by skin barrier and immunologic dysfunction. Viral and bacterial superinfection of cutaneous lesions, including eczema herpeticum and *Staphylococcus aureus* in patients with severe disease is well documented (Ong and Leung, 2016, Weidinger and Novak, 2016). Whether the general population of patients with AD has an increased risk of these and other types of infections because of an impaired skin barrier and/or immunologic dysfunction is unclear.

A recent meta-analysis of genome-wide association studies identified mutations in genes thought to play roles in the regulation of innate and adaptive immunity, in addition to established barrier function susceptibility loci such as filaggrin (Paternoster et al., 2015). Investigations of skin physiology suggest that differences in barrier function are identifiable very early in infancy and are highly predictive of the development of AD (Kelleher et al., 2015). We therefore hypothesized that individuals who develop AD are at increased risk of infections because of underlying genetically influenced immune and barrier dysfunction. The objective of our study was to determine if there was an association between AD and multiple common cutaneous and noncutaneous infections.

We performed a cohort study using The Health Improvement Network, a medical records database that is representative of the UK general population (Seminara et al., 2010). Ethics approval for this study was obtained from The Health Improvement Network Scientific Review Committee and the University of Pennsylvania Institutional Review Board. We included 3,112,617 individuals registered before age 18 years who were followed for a mean of 13.7 years (95% confidence interval = 13.6, 13.7). We identified subjects with AD based on the presence of at least one of any of the following diagnostic codes on two different visits, as is common practice in studies of chronic conditions using electronic health data (Herrett et al., 2010): atopic dermatitis and related conditions (M11.00), atopic dermatitis/AD (M111.00), and atopic dermatitis not otherwise specified (M11z.00). The prevalence of AD was 14.4% (95% confidence interval = 14.4–14.4).

We examined the prevalence of multiple common cutaneous and noncutaneous infections (warts, dermatophyte infection, impetigo, molluscum contagiosum, otitis media, pneumonia, and streptococcal throat infection; codes available in Supplementary Table S1 online). We found that all of the infectious illnesses we had determined to test a priori were more prevalent in those with AD. Using multilevel mixed-effects logistic regression, we examined the odds of each infectious outcome at any time point and found that the strength of association for cutaneous infections varied from a 55% increased odds of impetigo to a 3-fold increased odds of molluscum contagiosum after adjusting for sex, age, time of observation, and practice. Associations with noncutaneous infections varied from 27% increased odds of streptococcal throat infections to a 2-fold increase in otitis media (Table 1).

**Table 1:** Prevalence and risk of infectious outcomes among those with AD compared with those without AD

Variable	Descriptive Statistics		Primary Analysis: Risk of Infectious Outcome Ever		Sensitivity Analysis: Risk of Infectious Outcome Ever Using Different Exposure Definitions	
	Overall Prevalence (n = 3,112,617)	Prevalence among Those with AD (n = 448,311)	Crude	Adjusted^1^	Dermatitis (n = 632,707) Crude	AD and Asthma and/or Rhinitis (n = 162,116) Crude
	n % (95% CI)	n % (95% CI)	OR (95% CI)	OR (95% CI)	OR (95% CI)	OR (95% CI)
Cutaneous infections						
Cutaneous warts	285,011 9.12 (9.12–9.19)	72,681 16.21 (16.10–16.32)	2.23 (2.21–2.25)	1.98 (1.96–2.00)	1.50 (1.48–1.53)	2.91 (2.87–2.95)
Dermatophyte infection	24,693 0.79 (0.78–0.80)	7,899 1.76 (1.72–1.80)	2.83 (2.75–2.90)	2.54 (2.47–2.61)	2.14 (2.02–2.26)	3.56 (3.44–3.69)
Herpes simplex virus	65,027 2.09 (1.07–2.10)	18,461 4.11 (4.06–4.18)	2.41 (2.37–2.46)	2.08 (2.04–2.12)	1.81 (1.75–1.88)	3.26 (3.19–3.34)
Impetigo	21,467 0.69 (0.68–0.70)	7,899 1.76 (1.72–1.80)	3.50 (3.41–3.60)	1.55 (1.47–1.64)	2.06 (1.96–2.18)	3.82 (3.69–3.96)
Molluscum contagiosum	118,325 3.80 (3.78–3.82)	43,997 9.81 (9.73–9.90)	3.79 (3.74–3.84)	3.11 (3.07–3.14)	1.82 (1.78–1.86)	3.50 (3.44–3.56)
Systemic infections						
Otitis media	744,512 23.92 (23.87–23.97)	192,112 42.85 (42.71–43.00)	2.87 (2.85–2.88)	2.24 (2.22–2.25)	1.74 (1.72–1.76)	3.43 (3.40–3.47)
Pneumonia	69,880 2.24 (2.22–2.26)	17,087 3.81 (3.76–3.87)	1.96 (1.93–1.99)	1.27 (1.23–1.31)	1.48 (1.44–1.53)	2.76 (2.69–2.82)
Streptococcal throat infection	18,271 0.59 (0.58–0.59)	4,558 1.02 (0.99–1.05)	1.98 (1.92–2.05)	1.34 (1.26–1.42)	1.38 (1.30–1.46)	2.72 (2.60–2.84)

Abbreviations: CI, confidence interval; OR, odds ratio.

1 Adjusted for age at registration, sex, and time under observation. Physician practice included as a random effect in the model.

We performed sensitivity analyses exploring the definition of AD. When we estimated the association with a longer list of less-specific *dermatitis* codes (see Supplementary Table S1), we found that most associations were diminished. When we estimated the association with a more narrow designation, *AD plus asthma or seasonal rhinitis,* the magnitude of most of the associations increased. This highlighted a potential link between underlying immune dysfunction in atopic disease and increased susceptibility to infection.

Prior publications have found higher rates of infections among patients with AD, but most are from clinical populations, and therefore are likely to represent the more severe end of the AD spectrum (Beck et al., 2009, Peng et al., 2007), or are based on patient self-report, and therefore may be subject to recall and misclassification biases (Silverberg and Silverberg, 2014, Strom and Silverberg, 2016). Indirect evidence also comes from multiple studies that show an association between antibiotic use in early life and AD (Schmitt and Weidinger, 2014, Tsakok et al., 2013).

Strengths of this study include physician-confirmed diagnoses and a large longitudinal population-based sample. A number of potential limitations also warrant discussion. Our finding of an increased risk of cutaneous infections could be due to ascertainment bias (i.e., individuals with AD are more likely to have their skin checked and have skin conditions diagnosed). Although it is less likely that AD patients would have differential recording of systemic infections such as otitis or pneumonia, there may be a lower threshold for diagnosis or treatment of upper respiratory infections among patients with comorbid asthma, given the concern for asthma exacerbations with viral illness. Moreover, patients with chronic conditions like AD may be more likely to seek care. Nonetheless, our findings are important from a resource planning perspective; additional research is needed to understand the causal relationship between AD and infections. Finally, because AD can have a heterogeneous presentation, diagnosis may occasionally require specialist care, and we did not have access to dermatologist records. However, we believe our reliance on general practice physician records is reasonable in this context given that the vast majority (97%) of AD in the UK is treated in primary care (Emerson et al., 1998, Schofield et al., 2009).

We did not have detailed data about disease severity, flares, or timing of treatment use. Future studies examining whether there is a temporal association between these factors and infections could provide clinically useful prognostic information. Additionally, information on the timing of treatment use relative to infections could help establish whether specific treatment improves barrier function and reduces infection risk or whether immunosuppressive treatment increases infection risk. Because we studied the risk of infection at any time point (including before AD diagnosis and treatment), our results are unlikely to be confounded by immunosuppressive treatment use for AD. As noted, multiple studies have shown an association between AD and early-life antibiotic exposure, which provides support for our hypothesis that patients with AD are at an increased risk of infection even before AD diagnosis, and/or could indicate an effect of antibiotics on the development of AD (Schmitt and Weidinger, 2014, Tsakok et al., 2013). Additional work is needed to determine whether antibiotic treatment plays a causal role in the development of AD.

In summary, we found increased risks of all infectious outcomes examined, which include both cutaneous and noncutaneous infections caused by bacteria, viruses, and fungi. This observation raises numerous questions about the nature of immunological defects in AD. One study found that subjects with AD in whom eczema herpeticum develops have more severe T helper type 2 cell-polarized disease, more atopic comorbidities, and more cutaneous infections (Beck et al., 2009). Our study adds epidemiologic evidence suggesting that AD patients may additionally be at risk of noncutaneous infections. A significant body of functional and genotype/phenotype data has been developed for filaggrin (McAleer and Irvine, 2013), and there is a need for similar work linking immunological defects to clinical phenotypes.

Determining if individuals with AD are at increased risk of infections is important to guide the development of screening and prevention programs to reduce the morbidity associated with AD. Moreover, a baseline understanding of infectious risk is particularly important in the context of the introduction of the many new biologic therapies now in the pipeline for AD.

## ORCID

Sinéad M Langan: http://orcid.org/0000-0002-7022-7441

## Conflict of Interest

DJM is on separate data safety monitoring boards for Astellas, Janssen, Regeneron/Sanofi, and GlaxoSmithKline; the remaining authors state no conflict of interest.
