# Supplementary material for: Increased Risk of Cutaneous and Systemic Infections in Atopic Dermatitis—A Cohort Study
Source: J Invest Dermatol. 2017 Jun;137(6):1375–7. doi: 10.1016/j.jid.2017.01.030 (PMC5660507; doi:10.1016/j.jid.2017.01.030)
Supplement: Supplementary Table S1 [file mmc1.pdf]

SUPPLEMENTARY MATERIAL

Supplementary Table S1. Codes for exposure and outcomes

AD	Dermatitis	Cutaneous warts	Streptococcal throat infections	Pneumonia	Impetigo	Herpes simplex virus	Molluscum contagiousm	Allergic rhinitis	Otitis media	Asthma	Dermatophyte infections
M114.00	Myu2.00	7G09111	A340.00	4JUK.00	M05z.00	Q402000	43jb.00	14B1.00	F501G00	12D2.00	AB04.00
M113.00	Myu2200	A781400	A34..00	A022200		A54y.00	A780.00	1C83.13	F512100	14B4.00	AB04.12
M112.00	M114.00	A781.11	A340z00	A116.00		A544500	A780000	H00..16	F501C00	173A.00	
M111.00	M11..00	A781.00	A34z.00	A380300		A531z00		H120.00	F514000	173c.00	
M12z100	M11z.00	A781600	A340300	A3B2.00		9kF8.00		H120000	F501D00	178..00	
	M111.00	7G09011	A3B0.00	A3BXA00		A53y.00		H120100	F501411	1781	
	M119.00	A781000	A340100	A3BXB00		A54x.00		H120200	F502300	1782	
	M12z111	ZA3..00	A340200	A3C0300		A531000		H120300	F513111	1783	
	A540.00	8BAZ.11		A54x400		A532000		H120z00	F514300	1784	
	M12z100	7G06111		A551.00		A544100		H17..00	F502500	1785	
	F502411			A730.00		A53x.00		H17..11	F510300	1786	
	F4D3000			AB24.11		A53xz00		H170.00	F501B00	1787	
	M12z400			H06z100		A532z00		H170.11	F513000	1788	
	12H1.00			H06z112		A532100		H171.00	F513100	1789	
	M113.00			H2...00		A544z00		H171.14	F501y00	178A.00	
	14F1.00			H20..00		F030411		H172.00	F512z00	178B.00	
	M12z300			H20..11		A544000		H172.11	F522.00	1J70.00	
	M112.00			H200.00		A541400		H17z.00	F501E00	1O2..00	
	M12z200			H201.00		A531100		H330.13	F511100	2126200	
	M102.00			H202.00		A54x300		H330011	F502600	212G.00	
	M07y.11			H20y.00		A546.00		Hyu2000	F513z00	38DL.00	
	M102.11			H20z.00		65PQ.00			F510200	663..11	
	M101.12			H21..00		A541z00			F511300	663e.00	
				H21..11		9kF8.11			F513.00	663f.00	
				H22..00		A53x000			F514.00	663N100	
				H22..11		A544300			F524000	663N200	
				H220.00		A532300			F501z00	663O.00	
				H221.00		A541500			F511200	663O000	

H222.00	A548.00	F520100	663p.00
H222.11	A541000	F511.11	663P.00
H223.00	A54z.00	F521.00	663r.00
H223000	A544.00	F524.00	663t.00
H224.00	2524.00	F511z00	663v.00
H22y.00	A544200	F520000	663V100
H22y000	A54..11	F518.00	663V300
H22y011	2524.12	F502400	663w.00
H22y200	A54z.11	F520z00	663W.00
H22yX00	A541.00	F514z00	663x.00
H22yz00	A54..00	F512000	66Y5.00
H22z.00	A542.11	F510000	663h.00
H23..00		F512.00	663j.00
H23..11		F510z00	663m.00
H231.00		F525.00	663n.00
H232.00		F514100	663N.00
H233.00		F510011	663N000
H23z.00		F502700	663Q.00
H24..00		F511.00	663s.00
H240.00		F501100	663U.00
H241.00		F510100	663u.00
H242.00		F501F00	663V.00
H243.00		F514200	663V000
H243.11		F523.00	663V200
H246.00		F501000	663y.00
H247000		F512.12	66Y9.00
H247z00		F528.00	66YA.00
H24y.00		F520.00	66YC.00
H24y000		F512.11	66YE.00
H24y200		F52z.11	66YJ.00
H24y300		F526.00	66YK.00
H24y400		F527.00	66YP.00

		H24y600	F502.00	66Yp.00
		H24y700	F52..00	66YQ.00
		H24yz00	F510.00	66Yq.00
		H24z.00	F502z00	66Yr.00
		H25..00	F501.00	679J.00
		H25..11	F52z.00	66YR.00
		H26..00	F510500	66Ys.00
		H260.00		68C3.00
		H261.00		8CMA000
		H262.00		8CR0.00
		H270.00		8H2P.00
		H270.11		8791
		H270000		8793
		H270100		8794
		H270z00		8795
		H28..00		8796
		H2B..00		8797
		H2C..00		8798
		H2y..00		8HTT.00
		H2z..00		9N1d.00
		H470.11		9NNX.00
		H470312		9OJ..00
		H471000		9OJ..11
		H530300		9OJ1.00
		H540000		9OJ2.00
		H540100		9OJ3.00
		H564.00		9OJ4.00
		H564.11		9OJ5.00
		H56y000		9OJ6.00
		H56y100		9OJ7.00
		H571.00		9OJ8.00
		Hyu0800		9OJ9.00

		Hyu0A00	9OJA.00
		Hyu0B00	9OJA.11
		Hyu0D00	9OJZ.00
		SP13100	9Q21.00
